# Impaired Emotional Mirroring in Parkinson’s Disease—A Study on Brain Activation during Processing of Facial Expressions

**DOI:** 10.3389/fneur.2017.00682

**Published:** 2017-12-18

**Authors:** Anna Pohl, Silke Anders, Hong Chen, Harshal Jayeshkumar Patel, Julia Heller, Kathrin Reetz, Klaus Mathiak, Ferdinand Binkofski

**Affiliations:** ^1^Department of Psychology, University of Cologne, Cologne, Germany; ^2^Division of Clinical Cognitive Sciences, RWTH Aachen University, Aachen, Germany; ^3^Department of Neurology, University of Lübeck, Lübeck, Germany; ^4^Department of Neurology, RWTH Aachen University, Aachen, Germany; ^5^Institute of Neuroscience and Medicine (INM-4), Research Center Jülich GmbH, Jülich, Germany; ^6^Jülich Aachen Research Alliance (JARA), Translational Brain Medicine, Aachen, Germany; ^7^Department of Psychiatry, Psychotherapy, and Psychosomatics, RWTH Aachen University, Aachen, Germany

**Keywords:** neurodegeneration, mirror neurons, emotion, functional MRI, facial emotions, Parkinson’s disease

## Abstract

**Background:**

Affective dysfunctions are common in patients with Parkinson’s disease, but the underlying neurobiological deviations have rarely been examined. Parkinson’s disease is characterized by a loss of dopamine neurons in the substantia nigra resulting in impairment of motor and non-motor basal ganglia-cortical loops. Concerning emotional deficits, some studies provide evidence for altered brain processing in limbic- and lateral-orbitofrontal gating loops. In a second line of evidence, human premotor and inferior parietal homologs of mirror neuron areas were involved in processing and understanding of emotional facial expressions. We examined deviations in brain activation during processing of facial expressions in patients and related these to emotion recognition accuracy.

**Methods:**

13 patients and 13 healthy controls underwent an emotion recognition task and a functional magnetic resonance imaging (fMRI) measurement. In the Emotion Hexagon test, participants were presented with blends of two emotions and had to indicate which emotion best described the presented picture. Blended pictures with three levels of difficulty were included. During fMRI scanning, participants observed video clips depicting emotional, non-emotional, and neutral facial expressions or were asked to produce these facial expressions themselves.

**Results:**

Patients performed slightly worse in the emotion recognition task, but only when judging the most ambiguous facial expressions. Both groups activated inferior frontal and anterior inferior parietal homologs of mirror neuron areas during observation and execution of the emotional facial expressions. During observation, responses in the pars opercularis of the right inferior frontal gyrus, in the bilateral inferior parietal lobule and in the bilateral supplementary motor cortex were decreased in patients. Furthermore, in patients, activation of the right anterior inferior parietal lobule was positively related to accuracy in the emotion recognition task.

**Conclusion:**

Our data provide evidence for a contribution of human homologs of monkey mirror areas to the emotion recognition deficit in Parkinson’s disease.

## Introduction

Patients with idiopathic Parkinson’s disease (PD) suffer from a wide range of emotional disturbances including subjective feeling of emotions and the related physiological arousal states, but also recognizing and expressing them (1). These deficits are important as life quality is reduced and social interactions are hampered. Already, an early study suggested a negative influence of impaired facial expressiveness on interpersonal relationships (2), and psychosocial functioning was considered a key factor for health-related quality of life in PD [e.g., Ref. (3)]. Dopamine deficiency due to loss of nerve cells in the substantia nigra results in an imbalance of dopaminergic innervation in subcortico-cortical circuits, which causes typical motor and non-motor symptoms in PD (4). In these circuits, cortical and limbic areas are connected to different parts of the striatum, globus pallidus, substantia nigra, and thalamus (5).

Up to now, studies investigating emotion processing in PD have mostly focused on the orbitofrontal cortex (OFC) and the amygdala. A meta-analysis of structural imaging data revealed a reduction of left lateral OFC (BA47) gray matter volume in PD (6), which in turn was associated with disturbed facial emotion recognition (7). Furthermore, OFC activation has been found to be reduced in PD [left hemisphere (8), right hemisphere (9)] and in asymptomatic *Parkin* mutation carriers [left hemisphere (10)] during processing of affective facial expressions. It was assumed that diminished activation of the OFC is part of a dysregulation of the “mesolimbic gating loop” or the “lateral-orbitofrontal gating loop” (10). Pathological changes of the amygdala in PD involve the presence of Lewy bodies (11, 12), volume loss (7, 12), and a reduction of the dopamine binding level (13). Moreover, bilateral amygdala activation was found to be reduced in PD during an emotion discrimination task (14).

Another line of evidence supports the hypothesis that the link between emotion recognition deficits and facial motor impairments is closer than previously assumed. Reduced facial expressiveness is an important clinical symptom of PD, which might be useful for clinical evaluation of PD in the future [for a review, see Ref. (15)]. Three studies showed a significant relation voluntary control of facial muscles and of emotion recognition deficits (16–18). Response latencies during emotion recognition were shown to be negatively correlated with the amplitude of facial muscle responses in PD (19). Moreover, a relation between emotion recognition and a lack of automatic mimicry during observation of emotional facial expressions was recently reported (20). Although one study did not find a significant relation between velocity and amplitude of voluntary facial muscle activation and emotion recognition [note that sample size was small and effect size was not reported (21)], we assume that further examination of the association between emotion recognition deficits and facial motor impairments are valuable. Based on current theories of social cognition, it was assumed that the “neural resonance” in the observer’s motor system that normally facilitates understanding of facial expressions (22) is disturbed in PD (20). These theories are based on the detection of a special class of visuomotor neurons in inferior frontal area F5 [for a review, see Ref. (23)] and anterior inferior parietal area PFG of the macaque monkey (24–26). Area F5 is supposed to be the homolog of the human pars opercularis of the inferior frontal gyrus (IFGop) (27), area PFG the homolog of the human cytoarchitectonic area PFt, which is located in the supramarginal gyrus (28). Both areas are anatomically connected (29). The so-called “mirror neurons” were found to be activated not only during action execution but also during mere observation of goal directed actions and are thought to be a prerequisite for action understanding (30). It was assumed that observed facial expressions are transformed into motor and/or somatosensory representations, respectively, which help to understand the emotion (22). Overlapping activation during production or imitation and observation of emotional facial expressions was taken as evidence for the relevance of mirror neurons in the human IFGop and the anterior inferior parietal lobule (aIPL) for emotion processing (22, 31–34).

Up to now, the relation of emotion recognition and motor impairment in PD was examined in behavioral studies only. Here, we aimed at studying the neural substrate of these related disturbances. In a previous study, we showed that activation of right IFGop during execution and observation of pleasant facial expressions is increased in subclinical carriers of a single mutant *Parkin* allele. Importantly, increased activation in the IFGop was positively related to performance in an emotion recognition task. The *Parkin* allele mutation is a cause for early onset PD (35). It is accompanied by a reduction of the 18F-fluoro-L-DOPA metabolism (36), but mutation carriers do not suffer from clinical motor symptoms. It was assumed that the dopamine reduction is compensated by the motor system (37). In line with this, increased activation of the IFG related to improved emotion recognition was assumed to be compensatory (10). Based on this study, we examined whether patients with manifest idiopathic Parkinson’s disease show altered emotion recognition abilities and altered involvement of human homologs of mirror neuron areas and/or structures embedded in limbic- or lateral-orbitofrontal-basal ganglia circuits during execution and observation of emotional facial expressions. Impaired emotion recognition was postulated for the patient group. We mainly focused on activation of the right IFGop in manifest PD and assumed decreased activation due to a breakdown of the compensatory mechanism present in the subclinical stage in individuals with a Parkinson’s disease-associated mutation (10). Exploratory, we were also interested in potential activation differences in (i) bilateral aIPL, (ii) bilateral amygdala, and (iii) left OFC. Finally, we asked if altered brain activation was related to emotion recognition abilities in PD.

## Materials and Methods

### Procedure and Participants

Patients were recruited from the Movement Disorders Outpatient Clinic at the Department of Neurology and control participants were recruited in Aachen and the surrounding area. Before inclusion in the study, patients and volunteers were screened for mental disorders using a short German version of a DSM-IV interview [SKID-PIT light (38)]. Only volunteers with no self-reported history of mental illness were included in the study. 14 patients with Parkinson’ disease (PD) and 13 healthy controls (HC) underwent the study protocol. Data of one patient had to be excluded from analysis due to technical problems. Data of 13 PD (5f, 8m) and 13 age-matched HC with no self-reported history of neurological diseases (6f, 7m) were analyzed. All participants were right handed (39) and had normal or corrected-to-normal vision. PD were medicated with antiparkinsonian drugs according to their symptoms and measured in the “ON” state. The mean levodopa equivalent daily dose (LEDD) [calculated according to Ref. (40)] across all PD was 586.52 (*SD* = 494.88). The averaged disease duration for PD was 5.94 years (*SD* = 4.39, range 0.9–15.4 years). Patients with PD underwent the Mini-Mental-Status-Test (41). The averaged sum score was 28.31 (*SD* = 1.55; *Min* = 25). An experienced movement disorder specialist assessed motor dysfunction in PD using the motor subscale of the Unified Parkinson’s Disease Rating Scale [(UPDRS-III) (42) *M* = 24.21 (*N* = 12, SD = 9.60)].

Groups did not differ with regard to age [Median (*Mdn*)_HC_ = 65, *Mdn*_PD_ = 68; *U* = −0.13 *p* = 0.898], years of school education [*M*_HC_ = 10.92, SD_HC_ = 1.32, *M*_PD_ = 10.15, SD_PD_ = 1.68; *t*(24) = 1.3, *p* = 0.206] or crystallized intelligence, assessed by a German neuropsychological test [Mehrfachwahl-Wortschatztest version B MWT-B (43) *M*_HC_ = 118.62, *SD*_HC_ = 17.15, *M*_PD_ = 113.54, *SD*_PD_ = 20.17; *t*(24) = 0.69, *p* = 0.496].

### Experimental Setup

Participants underwent neuropsychological assessment and a functional magnetic resonance imaging (fMRI) measurement, completed a post-scanning questionnaire (PSQ) and a computer-based emotion recognition test.

We used the same experimental setup as in our study examining subclinical carriers of a single mutant *Parkin* allele (10) that was based on an earlier study on healthy participants (34). To assess emotion recognition, we used the Facial Expressions of Emotions—Stimuli and Test battery [FEEST (44)]. The FEEST contains two tests comprising photographs and morphed photographs of emotional facial expressions from the Pictures of Facial Affect series (45). These photographs are commonly used to estimate emotion recognition deficits in PD (46). In our study, participants underwent the Emotion Hexagon test. In this test, photographs of two emotions are blended, whereby the proportion of each emotion differs. Since sensing and expressing mixtures of emotions is very common (47), we assume a high ecological validity of the Emotion Hexagon test. The fMRI experiment enabled us to examine disturbances of the involvement of human homologs of mirror neuron areas in PD.

### Emotion Hexagon Test

Participants were asked to judge emotions in a six-alternative forced choice paradigm from morphed emotional facial expressions of different levels of difficulty (44). The FEEST consists of blended continua of two different emotional facial gestures, which are presented on a computer screen (45). Similar emotional expressions (happiness—surprise, surprise—fear, fear—sadness, sadness—disgust, disgust—anger, anger—happiness) are blended with different proportions of each emotion (90–10, 70–30, 50–50, 30–70, 10–90%) resulting in a total number of 30 morphs. The test contains 150 test trials split into five runs, in which, the 30 morphs were presented once in a randomized order. In total, each emotion occurred ten times in each degree of difficulty. The labels of the six basic emotions were provided at the bottom of the computer screen as alternative choice. Participants were asked to name the emotion that best described the facial expression displayed on the computer screen. Participants were familiarized to the task by 30 test trials prior to the actual test. Responses were entered into the computer by the experimenter. Responses to the 90–10 and the 70–30% morphs were defined correct if the predominant emotion was chosen by the participant. Responses to the 50–50% morphs were considered correct if one of the two emotions included in the morph was chosen by the participant.

### fMRI Experiment and Post-Scanning Rating Procedure

The stimulus material consisted of video clips, which had previously been used by us and our collaboration partners (10, 34, 48, 49). These clips consisted of emotional (smile), non-emotional (lip protrusion), and neutral stimuli (relaxed face without motion). The latter was used as a high level baseline. Each of the three facial expressions was shown by 24 actors (12 men), giving a total of 72 videos. Each video started with the actor displaying a neutral face for 1 s than expressing the facial gesture for 3 s and finally returning to display a neutral face for 1 s. Each video clip lasted 5 s in total. In addition, pixelated videos were created (Photoshop CS3 v.10.0^®^ and Adobe Premiere Pro CS3^®^). In these pixelated videos, the actors’ faces were scrambled into randomly moving squares, whereas the background remained original. A fixation cross either highlighted in red, blue, or green was presented on the scramble for the mid 3 s of the video to cue participants for execution of one of the three facial expressions (see below). The stimuli were presented with MR-compatible goggles (Resonance Technology, Inc., Northridge, CA, USA) using the Presentation© software package v.11.0 (Neurobehavioral Systems, Inc., Albany, CA, USA).

The fMRI experiment was planned as a 3 × 2 factorial design with factors “facial expression” (*emotional, non-emotional, neutral face*) and “task” (*execution, observation*). Participants were instructed to (i) attentively observe the videos displaying actors or (ii) in case of pixelated video clips to execute a facial expression. The kind of facial expression (emotional/smile, non-emotional/lip protrusion, neutral/relaxed face) was designated by the color of the fixation cross. The participants were to execute the facial expression as long as the fixation cross showed up. Participants’ facial expressions were filmed with a scanner-compatible camera and monitored online. The measurement was interjected in case of repeated errors (e.g., participant executed a wrong facial expression or mimicked the actor’s face during observation). The participant was then reinstructed and the run was restarted (this happened in case of two patients and one healthy control).

Immediately after the fMRI measurement, participants were asked to rate their subjective feeling of happiness during each condition (PSQ).

### Demographic and Behavioral Data Analyses

We analyzed behavioral data using IBM SPSS Statistics version 21. The proportional FEEST data were arcsine root square transformed before comparison of mean values by calculating a repeated measures ANOVA (50). Normal distribution of demographic and PSQ data was checked by evaluating skewness and kurtosis of these data in the two groups (51). A variable was assumed normally distributed when skewness [S = skewness/SE (skewness)] and kurtosis [*K* = kurtosis/SE (kurtosis)] were not greater than ±2. Because normality was assumed for the PSQ and most demographic variables, repeated measures ANOVAs and/or (*post hoc*) independent *t*-tests (*p* < 0.05, two-tailed) were calculated (see also Section “Results” and the Section “Procedure and Participants”). The assumption of normal distribution was violated concerning the age of participants. Therefore, a Mann–Whitney *U*-test was calculated to compare the age of the two groups (52). *Post hoc* pairwise comparisons were Bonferroni-corrected (*p* = 0.05/number of tests).

### fMRI Data Acquisition and Analyses

All fMRI data analyses were performed with SPM8 (Wellcome Department of Imaging Neuroscience, London, UK) implemented in Matlab 8.1 (Mathworks Inc., Sherborn, MA, USA).

### fMRI Data Acquisition, Preprocessing, and Single Subject Analyses

We obtained anatomical and functional images with a Siemens 3T Trio MR-scanner. Functional T2* weighted echo-planar images (EPIs) were obtained with the following parameters: TR = 2,400 ms, TE = 30 ms, flip angle = 90°, FoV = 240 mm, matrix size = 64 × 64, in-plane resolution = 3.8 mm × 3.8 mm, 36 slices with slice thickness 3.0 mm, and distance factor 3%. A high-resolution T1-weighted anatomical 3-D magnetization prepared rapid gradient echo image (TR = 1,900 ms, TE = 2.52 ms, TI = 900 ms, flip angle = 9°, FoV = 250 mm, 256 × 256 matrix, 176 slices per slab) was recorded.

The first five EPI volumes were discarded to allow for T1 equilibration effects. The remaining functional images were realigned to the first image to correct for head motion (53). Participants, except for one, moved less than 4.1 mm (translation) and 4.6° (rotation). One patient had a rotation value of 14°. Therefore, movement parameters were included as six additional regressors into the general linear model (GLM) as covariates of no interest to model variance related to absolute head motion. Prior to that, for each participant, the T1 image was co-registered to the mean image of the realigned functional images. The mean functional image was normalized to the MNI template [Montreal Neurological Institute (54, 55)], using a segmentation algorithm (56). Normalization parameters were applied to all EPI images and the T1 image. The images were resampled to 1.5 mm × 1.5 mm × 1.5 mm voxel size and spatially smoothed with an 8 mm full width half maximum isotropic Gaussian kernel.

Data were subsequently analyzed by a two-level approach. Using a GLM, each experimental condition (emotional observation, non-emotional observation, neutral observation, emotional execution, non-emotional execution, neutral execution) was modeled on the single-subject level with a separate regressor convolved with a canonical hemodynamic response function and its first temporal derivative. The parameter estimates for each voxel were calculated using maximum likelihood estimation and corrected for non-sphericity.

We calculated two second-level models, one to replicate previous findings on shared representations of facial expressions and one to identify brain activation differences of PD and HC. As a proof of concept, we identified shared representations for the emotional and the non-emotional facial expressions the current sample first. Following the analyses of our previous study in healthy participants (34), the neutral/static facial expression was conceptualized as high-level baseline. Activation during observation and execution of neutral facial expressions was subtracted on the single subject level: *Observation: emotional minus neutral facial expression* (E_OBS minus N_OBS) and *non-emotional minus neutral facial expression* (NE_OBS minus N_OBS); *execution: emotional minus neutral facial expression* (E_EXE minus N_EXE) and *non-emotional minus neutral facial expression* (NE_EXE minus N_EXE). Resulting four contrast images for each group (HC, PD) were fed into a flexible factorial second-level analysis using a one-way four-level ANOVA (factor: condition; blocking factor: subject). To identify shared representations for execution and observation, we calculated four conjoint conjunction analyses across the corresponding execution and observation contrast, separately for HC and PD and for the emotional and the non-emotional facial expression. In our previous study examining shared representations for happy and non-emotional facial expressions in healthy participants, we found significant differences between happy and non-emotional facial gestures, which we attributed to the enhanced communicative information of the emotional facial expression. We expected to replicate this previous finding here.

The second model was computed to assess differences between HC and PD. Here, we were especially interested in the communicative emotional facial gesture. All six first-level contrasts for each group (E_OBS, NE_OBS, N_OBS, E_EXE, NE_EXE, N_EXE) were fed into a flexible factorial analysis using a one-way 12-level ANOVA (factor: condition; blocking factor: subject). Four contrasts were computed: E_EXE: HC minus PD, HC minus PD; E_OBS: HC minus, HC minus PD. All contrasts were masked inclusive with OBS and EXE of the HC (*t* > 3.14) to restrict the analysis to brain regions activated during execution and observation, respectively, of emotional facial expressions in HC. Group comparisons were restricted to the emotional facial expression, but effects of interest plots were generated to display activation of areas of interest during all conditions. We obtained a mask, encompassing the right IFGop from the AAL atlas embedded in the WFU PickAtlas [Wake Forest University, Winsotn-Salem, NC, USA (57)] as IFGop was the region of most interest derived from the literature. Results of regions of interest analyses are reported FWE-corrected at a threshold of *p* < 0.05. Further exploratory whole brain analyses are reported at a threshold of *p* < 0.001, uncorrected, with an extent threshold of *k* > 10 voxel, to consider the small sample size. Brain structures were labeled using the Anatomy Toolbox v 1.6 (58, 59).

We extracted brain data from all clusters in the IFGop or IPL that were identified in the group comparison, because these two regions were the homologs of monkey mirror areas. We selected the eigenvariate option (adjusted for the effects of interest). Pearson’s correlation coefficient was used to assess the relation of brain data and emotion recognition in the FEEST as well as medication [LEDD (40)] and disease duration in PD, with results being reported significant at a threshold of *p* < 0.05 (two-tailed).

## Results

### Behavioral Data

Means and SDs of the PSQ and the emotion recognition accuracies in the FEEST are presented in Table 1.

**Table 1:** Post-scanning questionnaire (PSQ) and emotion recognition test [Facial Expressions of Emotions—Stimuli and Test battery (FEEST)].

	HC		PD patients	
PSQ	*N*	M (SD)	*N*	M (SD)
E_OBS	13	3.77 (1.69)	13	3.46 (1.71)
NE_OBS	13	2.92 (1.32)	13	3.00 (2.08)
N_OBS	13	2.23 (1.24)	12	2.67 (1.23)
E_EXE	13	4.15 (1.07)	13	4.15 (2.12)
NE_EXE	13	2.92 (1.55)	13	3.92 (2.06)
N_EXE	13	2.77 (1.64)	13	2.62 (1.89)

**FEEST**	**HC M (SD), ***N*** = 13**		**PD patients M (SD), ***N*** = 13**	
	**90–10%**	**70–30%**	**90–10%**	**70–30%**

Happiness	9.85 (0.38)	9.77 (0.6)	9.85 (0.38)	9.15 (1.14)
Surprise	8.15 (2.64)	7.77 (1.64)	8.54 (1.95)	7.77 (1.64)
Fear	8.0 (2.42)	7.31 (1.7)	7.00 (2.27)	5.23 (2.77)
Sadness	9.77 (0.6)	9.54 (0.66)	8.92 (1.38)	8.08 (2.53)
Disgust	7.13 (4.12)	6.85 (3.93)	7.38 (3.25)	6.69 (3.09)
Anger	8.46 (2.37)	7.85 (2.38)	8.77 (1.69)	7.92 (2.63)
50–50%	26.77 (3.0)		24.08 (3.35)	

*Means (M) and SD for the post-scanning questionnaire (top) and emotion recognition test (bottom)*.

*E_OBS, emotional observation; NE_OBS, non-emotional observation; N_OBS, neutral observation; E_EXE, emotional execution; NE_EXE, non-emotional execution; N_EXE, neutral execution; HC, healthy controls; PD, Parkinson’s disease; mean hits are displayed for the FEEST (90–10 and 70–30% condition consisted of 10 trials the; 50–50% condition consisted of 30 trials)*.

A 2 × 2 × 6 repeated measures ANOVA with within-subject factors “difficulty” (90–70 and 70–30%) and “emotion” (*happiness, surprise, fear, sadness, disgust, anger*) and between-subject factor “group” revealed a significant main effect of difficulty (*F*_1, 24_ = 20.30, *p* < 0.001) and a significant main effect of emotion (*F*_5, 120_ = 12.45, ε = 0.57, *p* < 0.001). There was neither a significant main effect of group (*F*_1, 24_ = 1.10, *p* = 0.306), nor any significant interaction effect (all *p* ≥ 0.328). A separate analysis was run for the 50–50% morphs because responses to these morphs were counted as correct if the participant recognized either of the two emotions contained in the morph. For the 50–50% morphs, PD performed significantly worse than HC (*t*_24_ = 2.43, *p* = 0.023).

Post-scanning ratings were analyzed with a repeated measures ANOVA with “facial expression” (emotional, non-emotional, neutral) and “task” (observe, execute) as within-subject factors and “group” as a between-subject factor. There was a significant main effect of facial expression (*F*_2,46_ = 12.79, *p* < 0.001) and a trend for the main effect task (*F*_1,23_ = 3.42, *p* = 0.077), but no significant main effect of group (*p* = 0.637) and no significant two or three-way interaction effect (all *p* > 0.32). Three *post hoc* tests were calculated resulting in a Bonferroni-corrected threshold *p* = 0.017. Participants rated their happiness higher after production and observation (values of both tasks were averaged) of emotional compared to non-emotional (*t*_25_ = 2.86, *p* = 0.008) or neutral facial expressions (*t*_25_ = 5.77, *p* < 0.001). The difference of happiness ratings between the non-emotional and neutral facial expression did not survive the Bonferroni corrected threshold of *p* = 0.017 (*t*_25_ = 2.38, *p* = 0.025).

### fMRI Data

In the fMRI results section, brain regions are labeled according to Anatomy Toolbox (58, 59) with cytoarchitectonic areas listed in brackets.

#### Manipulation Check: Shared Representations during Execution and Observation of Facial Expressions

**Figure 1:**
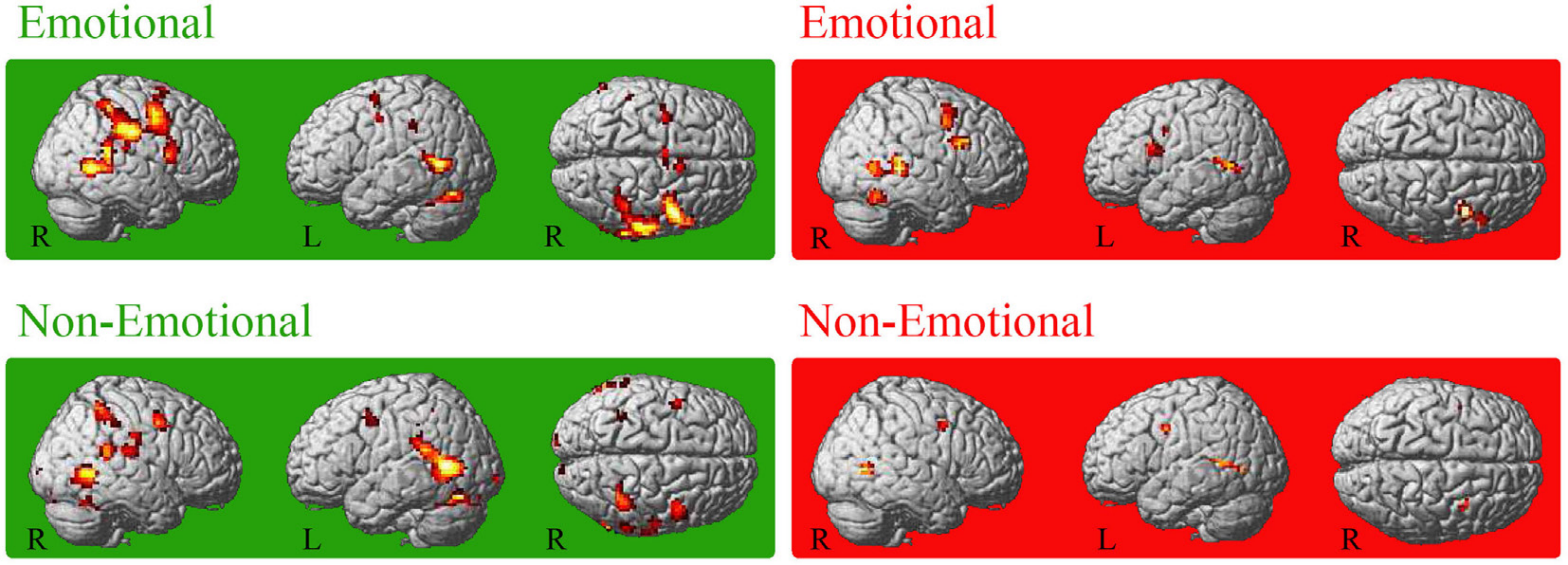
Conjoint activation for the observation and execution of facial expressions. Shared representations for observation and execution of facial expressions. Data of healthy controls are highlighted in green, data of patients with Parkinson’s disease in red. Abbreviations: HC, healthy controls; PD, patients with Parkinson’s disease; E_OBS, observation of emotional facial expressions; E_EXE, execution of emotional facial expressions; NE_OBS, observation of non-emotional facial expressions; NE_EXE, execution of non-emotional facial expressions. The conjunction is thresholded at *p* < 0.001 (uncorrected, *k* ≥ 10 voxel).

For both groups, conjoint conjunctions for execution and observation of the emotional facial expressions was found in bilateral precentral gyrus extending to right IFGop, right IPL (PF), right STG, and bilateral posterior middle temporal gyrus (MTG). HC additionally activated the bilateral (pre-)SMA, bilateral middle cingulate cortex, left supramarginal gyrus, right inferior temporal gyrus, right area PFt of the IPL and left cerebellum [VI (Hem), VIIa Crus I (Hem)] (see Figure 1). For patients, right frontal activation extended into the pars triangularis of the IFG and temporo-parietal activation extended into V5. Furthermore, right fusiform gyrus was activated in patients.

Conjoint activation during execution and observation of the non-emotional facial expression was found in the bilateral MTG in the conjunction analysis for each group. In HC, bilateral temporal cortex activation included also bilateral STG and right ITG. Furthermore, right IPL [intraparietal sulcus (IPS) hIP2, bilateral hIP3, supramarginal gyrus PF, PFm, PFcm, PFop], left posterior IPL, bilateral superior parietal lobule (SPL, 7PC), bilateral precentral gyrus (BA6), and bilateral cerebellum [VIIa Crus I (Hem), right VI (Hem)] were involved in HC. In PD patients, the conjunction revealed activation of the bilateral middle frontal gyrus (MFG), the bilateral posterior MTG activation extended area V5 (hOc5) and the left precentral gyrus (Table 2).

**Table 2:** Brain activation clusters for all whole brain contrasts.

		MNI							
Contrast		*X*	*Y*	*Z*	*k*	*t*-Value	*p*-Value	Side	Region
E_OBS ∩ E_EXE	HC	48	2	46	1,050	6.14	<0.001	r	Precentral gyrus
		−27	−73	−23	157	5.33	<0.001	l	Cerebellum
		−48	−58	4	166	5.16	<0.001	l	Middle temporal gyrus (MTG)
		−33	−4	64	15	4.41	<0.001	l	Precentral gyrus
		−45	−37	34	13	4.16	<0.001	l	Supramarginal gyrus
		−39	−7	46	19	3.88	<0.001	l	Precentral gyrus
		9	8	67	25	3.80	<0.001	r	Pre-supplementary motor area
		−6	−7	64	26	3.78	<0.001	b	Supplementary motor area
		−3	11	43	30	3.65	<0.001	b	Midcingulate cortex

E_OBS ∩ E_EXE	PD	45	−52	−20	66	4.92	<0.001	r	Fusiform gyrus
		45	11	25	85	4.55	<0.001	r	Inferior frontal gyrus pars opercularis
		42	5	43	59	4.55	<0.001	r	Precentral gyrus
		51	−61	7	157	4.31	<0.001	r	MTG
		−42	8	19	45	3.96	<0.001	l	Inferior frontal gyrus pars opercularis
		−54	−55	7	58	3.95	<0.001	l	MTG
		−39	2	37	11	3.74	<0.001	l	Precentral gyrus

NE_OBS ∩ NE_EXE	HC	−48	−58	4	572	6.89	<0.001	l	MTG
		36	−46	52	154	5.12	<0.001	r	Inferior parietal lobule (hiP3)
		51	−61	1	183	5.06	<0.001	r	MTG
		57	−40	19	75	4.90	<0.001	r	Superior temporal gyrus
		−27	−70	−20	260	4.77	<0.001	l	Cerebellum VI (Hem)
		63	−22	19	67	4.60	<0.001	r	Supramarginal gyrus
		42	−55	−26	36	4.52	<0.001	r	Cerebellum VI (Hem)
		45	2	43	67	4.21	<0.001	r	Precentral gyrus (BA 6)
		−45	2	46	25	4.16	<0.001	l	Precentral gyrus (BA 6)
		21	−85	−20	20	3.98	<0.001	l	Cerebellum VIIa (Hem)
		−12	−103	−5	15	3.71	<0.001	l	Calcarine gyrus
		−27	5	−23	15	3.66	<0.001	l	Temporal pole
		−30	−46	52	10	3.48	<0.001	l	Inferior parietal lobule (BA 2)
		15	−97	4	10	3.46	<0.001	r	Calcarine gyrus
		−9	−16	1	10	3.44	<0.001	l	Thalamus (prefrontal)

NE_OBS ∩ NE_EXE	PD	36	8	40	16	4.04	<0.001	r	Middle frontal gyrus (MFG)
		51	−64	4	16	3.94	<0.001	r	Middle temporal gyurs
		−45	−1	40	13	3.86	<0.001	l	Precentral gyrus
		−54	−52	7	34	3.80	<0.001	l	MTG

E_OBS^a^	HC > PD	12	−97	4	3,480	8.73	<0.001	b	Calcarine gyrus
		36	−49	49	515	7.48	<0.001	r	Inferior parietal lobule hiP3
		54	14	37	42	4.99	<0.001	r	Inferior frontal gyrus
		18	−25	−5	33	4.88	<0.001	r	Thalamus
		33	2	64	15	4.47	<0.001	r	Superior frontal gyrus
		−48	−25	40	11	4.13	<0.001	l	Inferior parietal lobule
		36	−7	40	24	4.00	<0.001	r	Precentral gyrus
		−21	−7	1	17	3.99	<0.001	l	Pallidum
		0	11	58	31	3.80	<0.001	b	Supplementary motor area
		27	−31	70	16	3.64	<0.001	r	Precentral gyrus
		48	47	4	10	3.57	<0.001	l	MFG
		−24	−22	4	18	3.54	<0.001	l	Thalamus (parietal, motor, somatosensory)

E_OBS^a^	PD > HC	–	–	–	–	–	–	–	–

E_EXE^a^	HC > PD	–	–	–	–	–	–	–	–

E_EXE^a^	PD > HC	24	−1	7	21	4.06	<0.001		Putamen

*MNI coordinates of the main peaks of significant clusters at a threshold of *p* < 0.001 uncorrected, the number of significant voxel (*k*), Table includes furthermore the hemisphere (L = left, R = right, and B = bilateral) of the cluster, and the name of the region in which the main peak was localized*.

*HC, healthy controls; PD, patients suffering Parkinson’s disease; E, emotional; NE, non-emotional; OBS, observation; EX, execution*.

*^a^All group comparison contrasts were masked inclusive with E_OBS and E_EXE of HC (*p* < 0.05)*.

#### Group Comparisons

**Figure 2:**
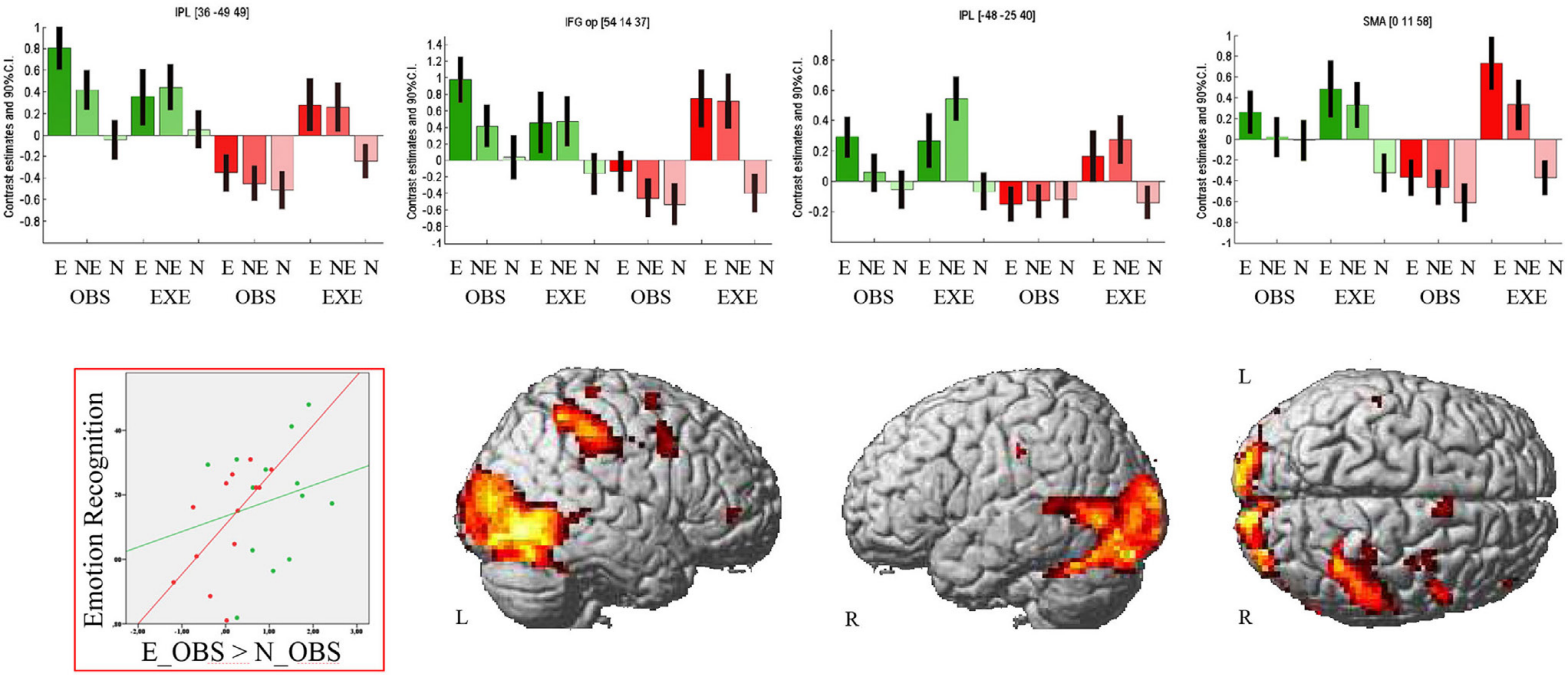
Observation of emotional facial expressions: Group comparison. Activation during observation of the emotional facial expression in healthy controls contrasted with activation during the same condition in patients with Parkinson’s disease. Bar graphs show brain activation level of human ‘mirror’ areas for all conditions: E, emotional; NE, non-emotional; N, neutral; OBS, observation; EXE, execution; healthy controls are colored in green, patients with Parkinson’s disease in red. The scatterplot depicts the relation of emotion recognition accuracy (Mean of 90–10% and 70–30% morphs, rPD(11) = 0.61, *p* = 0.026; rHC(11) = 0.2, *p* = 0.504) and activation of the right inferior parietal lobule (contrast E_OBS–N_OBS). The contrast is thresholded at *p* < 0.001 (uncorrected, *k* ≥ 10 voxel).

The region of interest analyses revealed decreased activation of the right IFGop during observation of the emotional facial expression in PD compared to HC [MNI (54 14 37), *t* = 4.99, *k* = 3, *p* < 0.001 FWE-corrected and MNI (54 17 19), *t* = 3.56, *k* = 3, *p* < 0.001 FWE-corrected]. Consequentially, decreased right IFGop activation was found in the exploratory whole brain analysis, too, but in this analysis extended to the right precentral gyrus. The exploratory whole brain analysis furthermore revealed stronger activation of right IPL (BA 1, BA2, BA40, PFt of the supramarginal gyrus, hIP3 of the IPS) and left IPL (BA2, PFt), bilateral (pre-) SMA, and bilateral thalamus in HC compared to PD during observation of the emotional facial expression (see Figure 2 and for a comprehensive enumeration, see Table 2).

Patients with PD did not show stronger activity than HC in any brain region in this condition.

Execution of emotional facial expressions resulted in increased activation of the right putamen in patients with PD compared to HC.

#### Correlation of Brain Activity, Emotion Recognition Accuracies, and LEDD Scores

In line with our hypotheses, we identified group differences in the IFG and IPL during observation of the emotional facial expression. We correlated brain activation in these regions during observation of the emotional facial expressions (E_OBS–N_OBS) with the emotion recognition accuracies for PD and HC separately. We used mean accuracy across difficulties (90–10, 70–30%) and across emotions as well as accuracy data concerning the 50–50% morphs. Finally, brain activation (E_OBS–N_OBS) was correlated with LEDD. In PD, we found a significant positive relation of right IPL activity and emotion recognition accuracy [*r*(11) = 0.61, *p* = 0.026] and a trend for a positive correlation of right IPL activity and emotion recognition concerning the ambiguous he 50–50% morphs (*r*(11) = 0.51, *p* = 0.077) in PD patients. No significant correlations were observed in HC (correlation of right IPL activity and mean emotion recognition: [*r*(11) = 0.2, *p* = 0.504]; and of right IPL activity and emotion recognition concerning the 50–50% morphs: [*r*(11) = 0.15, *p* = 0.62]). We found no significant correlation of right IFGop activity with mean accuracy [PD: *r*(11) = 0.4, *p* = 0.17; HC: *r*(11) = 0.25, *p* = 0.41] or with accuracy concerning the 50–50% morphs [PD: *r*(11) = 0.28, *p* = 0.35; HC: *r*(11) = 0.17, *p* = 0.59]. The same was true for the correlation of left IPL and mean accuracy [PD: *r*(11) = 0.4, *p* = 0.17; HC: *r*(11) = 0.3, *p* = 0.31] as well as accuracy concerning the 50–50% morphs [PD: *r*(11) = 0.2, *p* = 0.5; HC: *r*(11) = 0.14, *p* = 0.64]. There was no relation of medication and IFGop or IPL activity.

## Discussion

We examined emotion recognition abilities and underlying brain mechanisms in PD and HC. Both groups performed similarly well in the emotion recognition task, when ambiguousness of morphs was low. Because accuracy rates were high for 90/10 and 70/30% morphs, these morphs might have been too easy to recognize, to reveal group differences. In line with this assumption, patients had slightly more difficulties to recognize highly ambiguous facial expressions (50% morphs of two similar emotions). A meta-analysis on emotion recognition in PD revealed an impairment with a clinical significant medium but heterogeneous effect size in patients, with task difficulty assumed as possible influence factor (46). In line with this, FEEST tests had not the power to reveal deficits in a small sample of PD in a previous study (60), but differences were detected with a refined assessment of emotion recognition. As expected, both PD and HC activated inferior frontal and anterior inferior parietal human homologs of mirror neuron areas during execution and observation of emotional facial expression. As predicted, we found a significantly decreased response of fronto-parietal human homologs of mirror neuron areas in patients.

### IFGop

Both groups activated the right IFGop during execution and observation of emotional faces, but the response of the dorsal section of the IFGop and adjacent rostral part of the dorsal premotor cortex was reduced during observation of the emotional facial expression in PD relative to HC. Supposed to be the human homolog of monkey premotor mirror neuron area F5 (23), the IFGop has consistently been shown to be activated during observation and imitation of goal-directed movements (61) and processing of facial expressions (62). In our previous studies in healthy participants (34) and in *Parkin* mutation carriers (10), using a similar experimental design (but another scanning protocol and sample) conjoint activation for execution and observation of emotional facial expression was found inferior to the weaker than normal IFGop activation in PD in the current study. However, other studies found dorsal IFGop activated during observation (33, 63, 64) as well as during execution of emotional facial expressions (33) remarkably close to the peak in the present study. Activation of dorsal IFGop during observation of facial expressions was thought to represent activation of the mouth motor area (32) due to preparation of an automatic motor response, “facial mimicry” *or* a resonant activation of a dorsal sector of the mirror neuron system (33, 65, 66). Thus, reduced muscular mimicry accompanied by reduced resonance in the IFGop might account for emotion recognition deficits in PD. Besides this, lowered right dorsal IFGop activation was related with self-reported difficulties in identifying one’s own feelings (Alexithymia) in a recent study (67), and PD have been shown to suffer from Alexithymia double as often as control subjects (68). Thus, this region might also be important for the understanding of one’s own feelings. Interestingly, in contrast to Parkin mutation carriers (10), emotion recognition accuracy in PD with manifest Parkinson’s disease was not related to IFGop activity in the current study. Due to the small sample size, we had not enough power to detect small to medium size relations. Moreover, the FEEST might have been too easy to uncover emotion recognition deficits in PD. Future studies might use a more difficult emotion recognition test to enhance variance of emotion recognition accuracy and increase the sensitivity to detect relations between IFGop activation and behavioral performance.

### Parietal Cortex

We found widespread weaker than normal activation in patients with PD in the right IPL including supramarginal gyrus (PFt), somatosensory cortices, and the anterior IPS and a more localized decrease of activation in the left supramarginal gyrus (PFt) and somatosensory cortex.

Decreased activation of the supramarginal area PFt is in line with our hypothesis of a disturbed resonance in homologs of monkey mirror areas in PD, in contrast to stronger involvement of supramarginal gyri as shared representation of execution and observation of facial affect in HC [concerning HC, see also (69)]. Furthermore, somatosensory cortices [bilateral SI and SII (22), left SII (32), right SII (33)] have previously been shown to be activated during observation and execution/imitation of emotional facial expressions (but see two studies that did not find overlapping activity in the inferior parietal lobules 31, 34). In accordance with the positive relation of right IPL activation and emotion recognition accuracy in patients with PD in the current study, lesions of right IPL encompassing somatosensory areas and supramarginal gyrus have previously been reported to cause emotion recognition deficits (70). Furthermore, abnormal activation and connectivity patterns of the right IPL in patients with PD were shown in previous resting state studies (71, 72). Decreased connectivity of IPL and primary motor cortex and SMA was supposed to reflect a disturbance in networks linked to motor preparation and initiation in PD (73).

The IPS was assumed to be part of a frontoparietal network involved in adaptive online control of actions (74–76) and coding of action goals (75). Self- and other-generated actions are commonly represented in the IPS (75). Right IPS involvement was reported to be selective for face compared to object processing (62), shown during short presentation of facial expressions (77) and during emotion differentiation (78).

### Supplementary Motor Area

Analogously to our antecedent study (34), HC activated the right (pre-)SMA during observation and execution of emotional facial expressions. Interestingly, activation of this region was significantly decreased in PD patients. The Pre-SMA is involved in motor preparation (79). Activation during observation might, therefore, represent a starting contagious motor response of the observers face. Deviant activation in PD might result from functional changes in the basal ganglia-cortical motor loops, as pre-SMA is interconnected with the striatum (80) and the subthalamic nucleus (81), which in turn is connected to globus pallidus. The weaker activity in patients with PD compared to HC in our study is in accordance with a previously reported decreased brain activation (indexed through the amplitude of low frequency fluctuations) in the SMA during resting state (82) and an increase of activation during mental simulation of actions when PD patients were on compared to off dopaminergic medication (1).

### Limitations

We examined emotion recognition and processing of emotional facial expressions in a small sample of medicated patients with PD. Given the small sample size, the power of our study is appropriate to detect large correlations, but error probability β is high concerning medium and small effects (83). Furthermore, we measured medicated patients. And although our data are in accordance with and complement our findings in *Parkin* mutation carriers (10), and emotion recognition deficits in PD have been shown to be unrelated to dopaminergic medication (46), the results of this study have to be interpreted with caution until replication in larger samples and with unmedicated patients. Moreover, although similar brain activation patterns were shown during observation and execution of facial affect irrespective of which facial emotions were included (22, 32, 33), we cannot generalize our brain activation results to other emotions. Future studies could test the relation of emotion recognition and brain activation during observation of diverse facial emotions.

## Conclusion

We provide evidence for altered brain activation in manifest PD in human homologs of mirror neuron areas, which is partly linked to emotion recognition accuracy in PD. Visual input to the human mirror neuron system is supposed to be forwarded from posterior superior temporal sulcus to the parietal human homolog of monkey area PF/PFG where a motoric description is stored. Then, information is forwarded to IFGop and ventral premotor cortex, where action goals are coded (84). During imitation of actions, efference copies are sent back from frontal areas to IPL and from there to the posterior superior temporal sulcus. These efference copies allow a matching of sensory predictions of motor plans with the observed action (84). The transfer of information in this neural imitation circuitry might also be applicable to automatic facial mimicry. A disruption of information flow due to deactivation of frontal and parietal human homologs of mirror neuron areas in PD may lead to a disruption of neural resonance and thus be the basis of impaired emotion recognition.

## Ethics Statement

This study was carried out in accordance with the recommendations of the Declaration of Helsinki with written informed consent from all subjects. The study protocol was approved by the local Ethics Committee (Medical Faculty of the RWTH Aachen University; code: EK 099/08).

## Author Contributions

Author roles: (1) research project. (A) Conception: SA and AP conceptualized the study. AP developed the stimulus material and executed the rating and selection of stimuli. KM, FB, and AP were involved in applying for the ethics vote. AP and FB implemented the experiment for measurements with the fMRI scanner in Aachen. (B) Organization: FB and AP organized the study. KR and JH recruited patients, obtained medical history, and performed clinical examination. (C) Execution: AP screened participants, introduced them to study protocol, performed neuropsychological testing and a part of the fMRI measurements. HC and HP performed fMRI measurements and the emotion recognition task. (2) Statistical Analysis. (A) Design: AP and FB designed the data analyses. (B) Execution: AP, HC, and HP executed data analyses. JH calculated levodopa equivalence dose scores. (C) Review and critique: SA, KR, and KM reviewed statistics. (3) Manuscript preparation. (A) AP wrote the first draft. (B) All other authors reviewed the draft and approved the final version.

## Conflict of Interest Statement

The authors declare that the research was conducted in the absence of any commercial or financial relationships that could be construed as a potential conflict of interest.
